# DNA repair protein DNA-PK protects PC12 cells from oxidative stress-induced apoptosis involving AKT phosphorylation

**DOI:** 10.1007/s11033-021-06934-5

**Published:** 2021-11-19

**Authors:** Alessio Cardinale, Serena Saladini, Leonardo Lupacchini, Irene Ruspantini, Chiara De Dominicis, Marco Papale, Francesca Silvagno, Enrico Garaci, Cristiana Mollinari, Daniela Merlo

**Affiliations:** 1 Molecular and Cellular Neurobiology, IRCCS San Raffaele Roma, Via di Val Cannuta 247, 00166 Rome, Italy; 2 FAST. Istituto Superiore di Sanita’, Viale Regina Elena 299, 00161 Rome, Italy; 3 Department of Neuroscience, Istituto Superiore di Sanita’, Viale Regina Elena 299, 00161 Rome, Italy; 4 Department of Oncology, University Torino, via Santena 5 bis, 10126 Torino, Italy; 5 University San Raffaele, Via di Val Cannuta 247, 00166 Rome, Italy; 6 Institute of Translational Pharmacology, National Research Council, Via Fosso del Cavaliere 100, 00133 Rome, Italy

**Keywords:** DNA-PK, DNA damage, DNA repair, Oxidative stress, Apoptosis

## Abstract

**Background:**

Emerging evidence suggest that DNA-PK complex plays a role in the cellular response to oxidative stress, in addition to its function of double strand break (DSB) repair. In this study we evaluated whether DNA-PK participates in oxidative stress response and whether this role is independent of its function in DNA repair.

**Methods and results:**

We used a model of H_2_O_2_-induced DNA damage in PC12 cells (rat pheochromocytoma), a well-known neuronal tumor cell line. We found that H_2_O_2_ treatment of PC12 cells induces an increase in DNA-PK protein complex levels, along with an elevation of DNA damage, measured both by the formation of γΗ2ΑX foci, detected by immunofluorescence, and γH2AX levels detected by western blot analysis. After 24 h of cell recovery, γΗ2ΑX foci are repaired both in the absence and presence of DNA-PK kinase inhibitor NU7026, while an increase of apoptotic cells is observed when DNA-PK activity is inhibited, as revealed by counting pycnotic nuclei and confirmed by FACS analysis. Our results suggest a role of DNA-PK as an anti-apoptotic factor in proliferating PC12 cells under oxidative stress conditions. The anti-apoptotic role of DNA-PK is associated with AKT phosphorylation in Ser473. On the contrary, in differentiated PC12 cells, were the main pathway to repair DSBs is DNA-PK-mediated, the inhibition of DNA-PK activity causes an accumulation of DNA damage.

**Conclusions:**

Taken together, our results show that DNA-PK can protect cells from oxidative stress induced-apoptosis independently from its function of DSB repair enzyme.

**Graphical Abstract:**

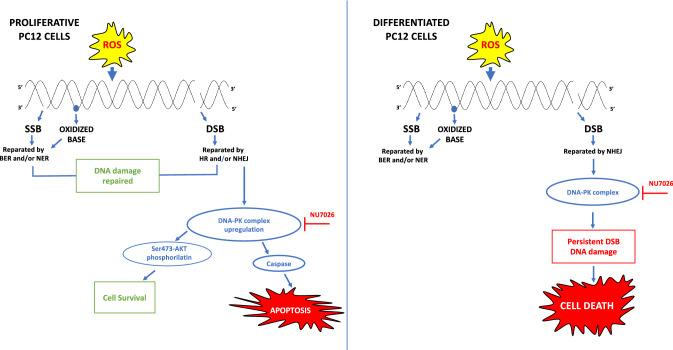

## Introduction

Oxidative stress induces DNA damage, and the unrepaired or improperly repaired DNA lesions increase genomic instability, which can cause cell death, senescence, or dysregulation of cellular functions. Oxidative stress can induce cellular damage by Reactive Oxygen Species (ROS) generation, which are constantly produced inside the cell and able to cause modifications or alterations of DNA with serious consequences especially for neuronal cells that last throughout life [1–3]. DNA damages induce and coordinate a complex signal-transduction network composed by several pathway activation, collectively named DNA Damage Response (DDR), which can detect DNA lesions and arrest the cell cycle or promote cell death (apoptosis) in case of severe and irreparable damage [4–6]. In mammalian cells, the most severe form of DNA damage (double strand breaks, DSBs) is repaired by non-homologous end joining (NHEJ) and homologous recombination (HR) [7, 8]. NHEJ is considered the prevalent DSB repair pathway operating in neurons, which relies on the DNA-dependent protein kinase (DNA-PK) complex [9].

DNA-PK is a PI3 kinase family member (which includes also Ataxia telangiectasia and Rad3-related, ATR and Ataxia-telangiectasia mutated kinase, ATM) preferentially phosphorylating serine and threonine residues followed by a glutamine, although other S-T/hydrophobic residues are also phosphorylated [10–12]. The active protein is a trimeric complex composed of the catalytic subunit, DNA-dependent protein kinase catalytic subunit (DNA-PKcs), and the Ku70/86 heterodimer which binds to DNA ends with very high affinity and functions as regulatory subunit that stimulates DNA-PKcs kinase activity [13]. Moreover, the Ku subunits have been implicated in the cellular response to oxidative stress [14]. In addition to its role in DNA DSB repair, DNA-PK has been involved in several pathways including stress response modulation, apoptosis, telomere homeostasis and specific gene transcription [15]. The other members of PI3 kinase family, such as ATM, also has been involved in oxidative stress response and can be directly activated by hydrogen peroxide (H_2_O_2_) [16, 17]. Indeed ROS over-production leads to rapid ATM dimerization/activation and downstream ATM signalling thus modulating cellular metabolism and cell survival, repairing oxidative DNA damage, and inducing antioxidant enzyme expression to maintain redox homeostasis [16, 18]. ROS can also activate DNA-PK and its downstream signalling similar to ATM [19]. Indeed, DNA-PK has been found to associate with base excision repair (BER) protein complex essential for removing oxidative base damage [20]. Among the BER components, X-ray repair cross-complementing protein 1 (XRCC1) directly interacts with and is phosphorylated by DNA-PK [21]. In addition, Peddi et al. reported that DNA-PKcs deficiency compromises BER activity and inhibits the efficient processing of DNA lesions induced upon IR or H_2_O_2_ treatment [22]. Although recent evidences have implicated a role of DNA-PK in oxidative stress response [19], the molecular mechanism by which DNA-PK functions in the oxidative stress response remains to be elucidated.

In this study we evaluated whether DNA-PK participates in oxidative stress response and whether this role is independent of its function of DNA repair. To test this hypothesis, we used a H_2_O_2_-mediated oxidative stress model in rat pheochromocytoma line 12 (PC12) cells, a well-known neuronal cell line, to study the effect of oxidative stress on DNA-PK complex expression levels and function.

Our data clearly demonstrate that the DNA-PK complex expression and activity are stimulated by oxidative stress. Enhanced apoptosis in the presence of DNA-PK kinase inhibitor provides evidence that its role of reparative DNA enzyme is disjuncted by its antiapoptotic role. Indeed, we found that accumulated Histone H2AX phosphorylation (γH2AX) foci are repaired after 24 h recovery both in the absence and presence of DNA-PK inhibitor.

In conclusion, our study indicates that DNA-PK may have a crucial role in cellular oxidative stress response and the enhancement of its activity may open new perspectives for the treatment of ROS-related diseases.

## Materials and methods

### Cell culture and treatments

PC12 cells were a kind gift from Prof. Silvia Biocca, Università degli Studi di Roma Tor Vergata, Dipartimento di Medicina dei Sistemi (original source: LA Green’s lab [23].

PC12 cells were cultured and passaged as previously described [24]. Briefly, PC12 cells were cultured in RPMI 1640 medium (invitrogen) supplemented with 10% horse serum (HS, Euroclone), 5% fetal bovine serum (Euroclone), 2 mM L-glutamine (BioWest), 100 units/ml penicillin and 100 µg/ml streptomycin (BioWest). The cells were cultured on 100-mm/35 mm-diameter tissue culture dishes (Falcon™, BD Biosciences) and maintained in a 37 °C incubator in a water-saturated, 5% CO2 atmosphere. When the cultured cells reached 80–90% confluency (split ratio 1:4), they were detached by trypsinization (0.025% trypsin-EDTA) and sub-cultured. Cells were centrifuged and harvested after trypsinization (0.025% trypsin-EDTA).

To induce neuronal differentiation, PC12 cells were cultured on Matrigel in RPMI-1640 medium supplemented with 5% HS, 2% FBS and penicillin-streptomycin containing 100 ng/ml NGF (mouse nerve growth factor 2.5 S grade I, Alomone Labs) for 7-9 days. Medium was replaced every 2 days.

Different concentrations of H_2_O_2_ were tested in the oxidative stress injury model according to the previous studies [25, 26]. Cell viability was assessed to determine the optimal H_2_O_2_ concentration. In our model, 0.3 mM H_2_O_2_ was considered the best concentration for the oxidative experiments. DNA-PK inhibitor NU7026 (Calbiochem) was diluted at 7.1 mM and used for cell treatment at final concentration 10 µΜ. Insulin was purchased from Sigma-Aldrich and used at the final concentration of 100 nM for different incubation times (10–30 min).

We routinely checked our cell cultures and confirm it to be free of mycoplasma contamination by using “MycoStrip mycoplasma detection kit from Invivoen.

### Western blot analysis

Protein extracts derived from cultures were subjected to determination of protein concentration by using the bicinchoninic acid kit (Micro BCA, Pierce). Appropriate amount of protein extracts was boiled for 5 min in SDS-PAGE Laemmli buffer (50 mM Tris-HCl, pH 6.8, 2% SDS, 10% glycerol, 0.1% bromophenol blue, 50 mM DTT) and separated by SDS-PAGE (5% polyacrylamide for DNA-PKcs, 10% for Ku70, Ku86, AKT β-Actin and β-tubulin). Proteins were electrotransferred onto nitrocellulose membrane (HybondTM C-extra, Amersham Biosciences, UK limited) at 30 V overnight at 4 °C for DNA-PKcs and 100 V for 1 h at 4 °C for the other analyzed proteins. Membranes were blocked for 1 h at room temperature (RT) with 10% (w/v) milk in TBS-T solution (blocking buffer, 0.1% Tween20 in 1.3 M NaCl, 200 mM KCl, 250 mM Tris-HCl, pH 7.5) and incubated overnight at 4 °C with primary antibodies and then with the appropriate horseradish peroxidase-conjugated secondary antibody for 1 h at RT. Immunoreactive bands were detected by enhanced chemiluminescence detection system (EuroClone). The following primary antibodies were used: mouse DNA-PKcs Ab-4 mixture 1:400 (NeoMarkers MS-423-P); goat anti Ku70 1:400 (Santa Cruz Biotechnology sc-1486); goat anti Ku86 1:500 (Santa Cruz Biotehnology sc-1484); Rabbit anti total AKT 1:1000 (Cell Signaling CST-9272); rabbit anti pospho Ser-473 AKT 1:1000 (Cell Signaling CST-9271 S); rabbit anti pospho Thr-308 AKT 1:1000 (Cell Signaling CST-9275 S); mouse anti anti-β-Actin 1:1000 (Sigma A3853); mouse anti β-tubulin 1:1000 (Sigma T8328); mouse anti γH2AX antibody 1:1000 (Millipore (Ser139), clone JBW301); rabbit anti-Caspase3 1:1000 (Cell Signaling CST-9662); rabbit anti-PARP-1 1:1000 (Cell Signaling CST-9542); rabbit anti total ERK 1:1000 (Cell Signaling CST-9102) and rabbit anti phospho-ERK (p42 and p44) 1:1000 (Cell Signaling CST-9101) in TBS-T containing 2 or 5% milk for 2 h or overnight at 4 °C with gentle shaking. After extensive washing in TBS-T, membranes were probed for 1 h at RT with Horseradish peroxidase-conjugated antibodies donkey anti-mouse IgG 1:100,000 (715-035-151) and anti-rabbit IgG 1:100,000 (711-035-152) (Jackson ImmunoResearch), anti-goat IgG 1:500,000 (sc-2768) (Santa Cruz Biotechnology) were used as secondary antibodies, diluited in TBS-T containing 2% milk and washed thoroughly with TBS-T. Blots were scanned and densitometric analysis was performed by using ImageQuant software (GE Healthcare). Protein loading was monitored by normalization to anti-β-Actin or β-Tubulin.

### Immunofluorescence analysis

Immunofluorescence analysis was performed on proliferating and differentiated PC12 cells grown on matrigel-coated glass coverslips, fixed with phosphate buffer containing 4% paraformaldehyde and permeabilized with 0.2% Triton X-100 to then be processed for immunofluorescence analysis according to [27]. Anti MAP2 antibody (1:500, Sigma) and mouse anti γH2AX antibody 1:500 (Millipore (Ser139), clone JBW301) were used as primary antibodies by incubation for 1 h at 37 °C in 1× PBS containing 0.05% Tween 20 and 3% BSA, followed by a 30 min incubation at 37 °C with the secondary antibodies (Alexa 488, Alexa 568, Molecular Probes). Nuclei were counterstained with Hoechst and samples were mounted on glass slides and cover slipped with antifade glycerol mounting. For apoptotic cell detection, picnotic nuclei were counted and the percentage was calculated on the total cell number/field (10× objective) [28]. Images were acquired with an Eclipse 80i Nikon Fluorescence Microscope (Nikon Instruments, Amsterdam, Netherlands).

### Cell cycle and apoptosis by FACS analysis

For cell cycle analysis PC12 cells were mechanically dissociated and resuspended in Nicoletti’s buffer, containing 0.1% Sodium Citrate, 10 mM NaCl, 0.1% Triton X-100, 200 mg/mL Propidium Iodide (PI) and 200 mg/mL RNAse A [29]. Following 30 min incubation at room temperature cells were acquired with a FACSCanto flow cytometer (BD Biosciences).

### Statistical analysis

Statistical analysis was conducted in R language (R Core Team (2020). R: A language and environment for statistical computing. R Foundation for Statistical Computing, Vienna, Austria. URL https://www.R-project.org/). As a first step, normality and homoscedasticity were assessed using Shapiro-Wilk test (R function: shapiro.test()) and Levene’s (R function: leveneTest(), “car” package), respectively. The assumptions showed to be met in our data set. Successively, significant differences between groups were evaluated with unpaired two-tailed t-test, one-way ANOVA and two-way ANOVA with interaction, depending on the specific experimental design as reported in the “Results” section. In case of significant omnibus tests, Tukey’s Honestly-Significant-Difference (R function: TukeyHSD()) was applied for performing multiple pairwise-comparison between the means of groups. When two-way analyses were conducted and interaction was significant, main effects were not reported, whereas the complete post-hoc test results were shown in the figures. A significant difference was accepted at p-values below 0.05.

## Results

### H_2_O_2_ treatment of PC12 cells induces up-regulation of DNA-PK complex expression

H_2_O_2_ is known to increase ROS production in cells, often leading to apoptosis and cell death [30, 31]. It has been shown that oxidative damage can cause nuclear and mitochondrial DNA damage, and can modulate expression of repair enzymes in neuronal cells [32, 33]. DNA-PK complex kinase activity is regulated by different mechanisms, including modification in protein levels of the catalytic subunit DNA-PKcs and/or the regulatory subunits Ku70 and Ku86 [34–36].

**Fig. 1:**
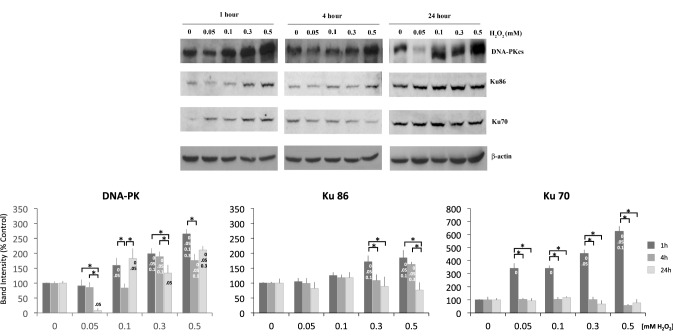
Representative western blots of DNA-PKcs complex in PC12 cells treated for 1, 4, and 24 h with different mM doses of H_2_O_2_. After H_2_O_2_ incubation, cells were processed to obtain whole cellular extracts as described in “Materials and Methods” section and DNA-PKcs, Ku86 and Ku70 protein levels were assayed by western blot analysis. β-Actin was used as loading control. Densitometric quantitation of the immunoreactive bands corresponding to DNA-PKcs, Ku70 and Ku86 are represented in plots. Values in plots represent the normalized percent changes in protein levels with respect to control (100%) after exposure to H_2_O_2_. Results were representative of 5 independent experiments. [*] Significant differences (p < 0.05) between time points within each concentration. [Concentration labels on bars] Significant differences (p < 0.05) between concentrations (0, 0.05, 0.1, 0.3, and 0.5) at the same time point, i.e. conc. labels are reported on a bar when a contrast between that group and any on its left is significant

To evaluate whether H_2_O_2_ can modulate DNA-PKcs protein levels in PC12 cells thus influencing DNA repair, we first treated proliferating PC12 cells with H_2_O_2_ at different concentrations and at different times. PC12 cells were exposed to concentrations of H_2_O_2_ in a range of 0.05–0.5 mM for 1 h, 4, and 24 h and then western blot analysis was performed on whole cell extracts. A two-way ANOVA showed a significant interaction between H_2_O_2_ concentrations and exposure time [F(8, 45) = 17.75, p < 0.001], thus meaning that DNA-PKcs protein levels were up-regulated in a H_2_O_2_ concentration-dependent manner with different profiles over time (Fig. 1). Particularly, post-hoc tests showed that 1 h exposure determines an increase of DNA-PKcs protein levels as H_2_O_2_ concentration grows; a step-like behavior was observed after 4 h exposure, with a plateau at higher H_2_O_2_ concentrations; for the 24 h-treatment DNA-PKcs levels varied in a H_2_O_2_ concentration independent manner. Indeed, we observed a strong reduction (90%) of DNA-PKcs levels at 24 h treatment with 0.05 mM which was unexpected and inexplicably reproducible (Fig. 1). Moreover, 0.5 mM H_2_O_2_ treatment yielded the greatest effect both in acute (1 h) and chronic treatment (24 h) (250%, and 220% increase respectively), differently from 4 h-treatment that reached its maximum (189%) already at 0.3 mM H_2_O_2_. We then analyzed whether treatment of PC12 cells with H_2_O_2_ was able to modulate protein levels of the DNA-PK complex regulatory subunits, Ku70 and Ku86. In both cases we found a significant interaction between H_2_O_2_ concentrations and exposure time [Ku 86: F(8, 45) = 8.50, p < 0.001; Ku 70: F(8, 45) = 101.13, p < 0.001]. Densitometric analysis of immunoreactive bands (Fig. 1) showed that only a short treatment (1 h) could increase levels of both proteins in a H_2_O_2_ dose dependent manner.

These findings support the hypothesis that DNA-PK expression is induced rapidly after DNA damage with a mechanism that needs further investigation.

### H_2_O_2_ treatment induces DNA damage in PC12 cells which is repaired after recovery

**Fig. 2:**
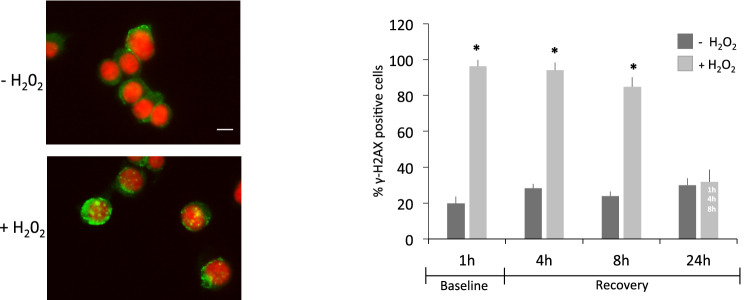
Left Panel. Immunofluorescence images of proliferating PC12 cells in the absence or presence of 0.3 mM H_2_O_2_ treatment. Only cells exposed to H_2_O_2_ treatment show γH2AX positive foci (green) in nuclei counterstained with Hoechst (red). Scale bar, 5 μm. Right Panel. After exposure with 0.3 mM H_2_O_2_ for 1 h (baseline), PC12 cells were incubated for 4, 8, and 24 h with fresh H_2_O_2_-free medium (recovery condition). Cells were fixed and stained with anti-γH2AX antibody and subjected to immunofluorescence microscopy. The number of γH2AX-positive cells were counted and plotted as histograms to show the repair kinetics of damaged DNA. After 24 h recovery, DNA damages are completely repaired. Results were representative of 5 independent experiments. [*] Significant differences (p < 0.05) between +/− H_2_O_2_ conditions at each time point. [Time labels on bars] Significant differences (p < 0.05) between time points (1 h, 4 h, 8 h, and 24 h) in the same H_2_O_2_ condition, i.e. time labels are reported on a bar when a contrast between that group and any on its left is significant. (Color figure online)

To analyze whether the upregulation of DNA-PK complex was associated with repairing of H_2_O_2_-mediated DNA damage, we chose the optimal dose of 0.3 mM as the smallest dose inducing DNA-PK complex protein level increment (200% increase, 1 h treatment). We then evaluated the DNA damage and repair kinetics by monitoring the formation and disappearance of γH2AX foci, a well-known and sensitive molecular marker of DNA damage, using immunofluorescence (Fig. 2). Cells having more than 10 foci/nucleus were scored positive. Left panel shows a representative immunofluorescence of γH2AX foci formation after 0.3 mM of H_2_O_2_ 1 h treatment.

We then performed a kinetics of γH2AX dephosphorylation at different times after H_2_O_2_ removal, up to 24 h, in which cells were maintained in fresh growing medium (condition defined as “Recovery” time). As it would be expected, a significant interaction was found between the effect of damage and the effect of exposure time on the amount of γH2AX positive cells [F(3, 24)=119.45, p < 0.01] (Fig. 2, right panel). Post hoc tests revealed that 0.3 mM H_2_O_2_ was able to induce a significant 75% increase (p < 0.001) of γH2AX positive cells as compared to control, 1 h after treatment (baseline). Successively, during the post-treatment repair time (Recovery), the percentage of γH2AX foci-positive cells gradually declined reaching the same levels as the untreated control cells 24 h after recovery (30% of γH2AX foci positive cells, p = 0.99).

These results may suggest that after exposure to an oxidative damage, cells may respond by up-regulating DNA repair enzymes to overcome the injury.

### Inhibition of DNA-PK kinase activity increases apoptosis without affecting DNA repair in proliferating PC12 cells

**Fig. 3:**
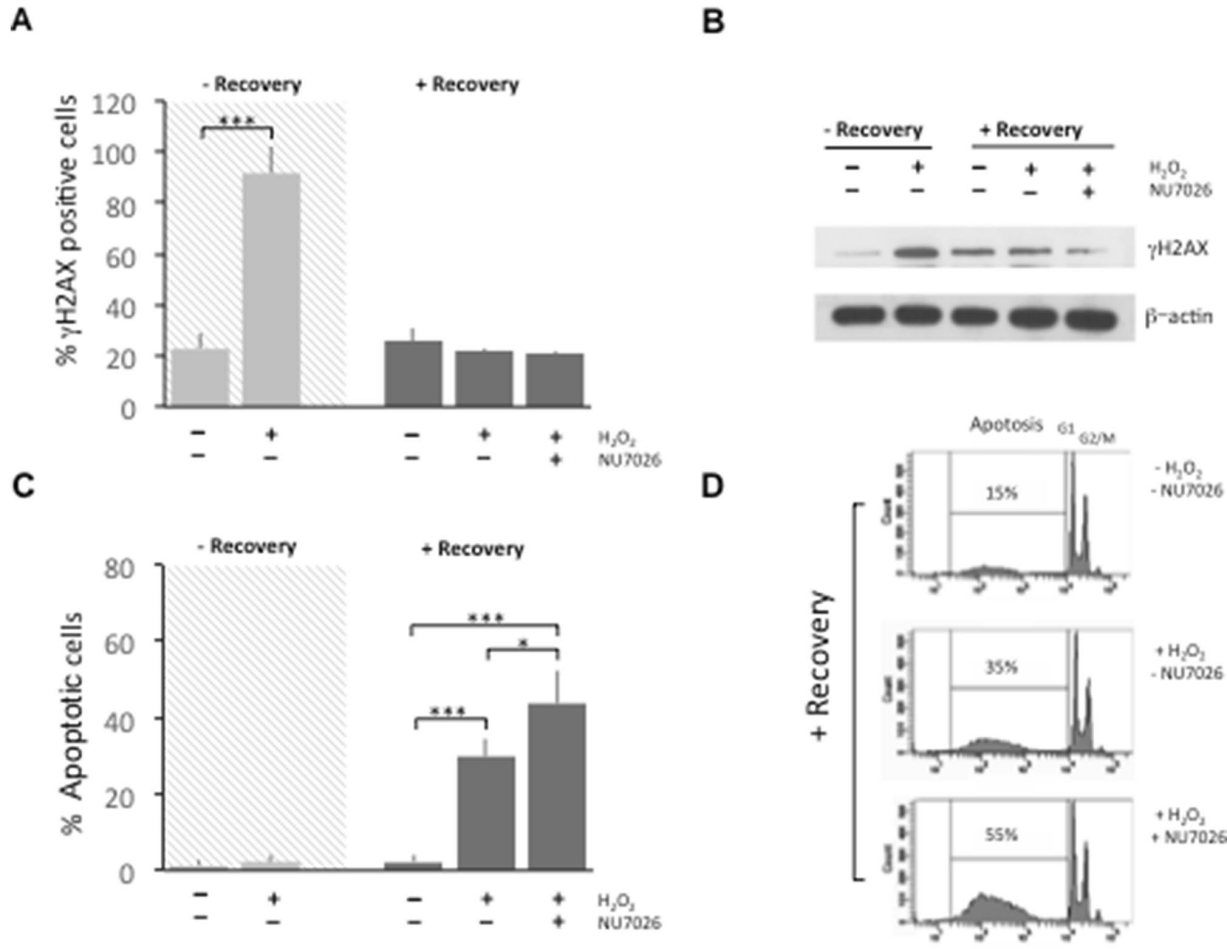
PC12 cells were treated for 1 h with 0.3 mM H_2_O_2_ and then incubated for 24 h with fresh medium with or without NU7026 (10 µM), a potent DNA-PK inhibitor. Cells were fixed and stained with anti-γH2AX antibody to count foci and nuclei were counterstained with Hoechst to count condensed and/or fragmented nuclei as apoptotic cells. **A** Bar chart showing that γH2AX foci are repaired after a 24 h recovery even if DNA-PK activity is inhibited. As supplementary information, percentage foci without recovery (grey background) are reported to show the increase after H_2_O_2_ treatment. **B** Representative western blots of γH2AX in PC12 cells treated for 1 h with 0.3 mM of H_2_O_2_ confirming the repair of DNA damage both in presence and absence of DNA-PK inhibitor during recovery. After H_2_O_2_ incubation, cells were processed to obtain whole cell extracts as described in “Materials and Methods” section. β-actin was used as loading control. Image is representative of 3 independent experiments. **C** Bar chart showing that 0.3 mM H_2_O_2_ treatment caused a 30% of apoptotic cells after 24 h recovery. A further increase is observed in the presence of 10 µM NU7026, as compared with H_2_O_2_-treated cells (+15%). In addition, without repair (grey background) H_2_O_2_ treatment did not induce augmented apoptosis (p = 38). Bars in the plot represent mean ± S.D. of apoptotic cells expressed as percentage. **D** FACS analysis was conducted to confirm the occurrence of apoptosis after 0.3 mM H_2_O_2_ 1 h treatment followed by 24 h recovery both in the presence ad absence of 10 µM NU7026. Cells were stained with PI, according to Nicoletti’s protocol. Histograms show DNA content distribution in the different experimental conditions and indicate an increase of approx. 20% in apoptotic cells in the presence of DNA-PK inhibitor. Apoptotic cells appear with fractional DNA content before the peak of G1 cells

To establish whether DNA-PK complex kinase activity has a role in DNA repair following H_2_O_2_, we performed experiments in presence of NU7026 (10 µM), a specific DNA-PK kinase inhibitor. Figure 3A shows that after 0.3 mM H_2_O_2_ treatment, PC12 cells accumulate γH2AX foci (69%, t(6)=11.45, p < 0.001). After 24 h recovery, differences in means between untreated, H_2_O_2_-treated, NU7026-added cells were not significant [F(3, 9) = 3.059, p = 0.096]. PC12 cells repaired foci both in the absence and presence of NU7026 inhibitor, indicating that other DNA-PK independent repair mechanisms may be operative in proliferating PC12 cells. Western blot analysis of protein levels of γH2AX confirmed the presence of DNA damage following 1 h 0.3 mM H_2_O_2_ treatment, that decreased after recovery both in absence and presence of DNA-PK inhibitor (Fig. 3B).

We also evaluated the percentage of apoptotic cells after 0.3 mM H_2_O_2_ 1 h treatment (Fig. 3C). After 24 h recovery, the percentage of apoptotic cells significantly varied with cell conditions [F(3, 9) = 52.74, p < 0.001]; pairwise comparisons were thus evaluated with post hoc tests. Figure 3C shows that H_2_O_2_ treatment caused a 30% of apoptotic cells (p < 0.001) during recovery. This amount further increased in the presence of 10 µM NU7026 by approx. 15% as compared with H_2_O_2_-treated cells (p = 0.019). This result was surprising because, in the same conditions, DNA damage was repaired as indicated by the return to basal level of γH2AX foci (Fig. 3A) and suggests a protective role of DNA-PK under oxidative stress conditions.

In addition, we confirmed the occurrence of apoptosis after 0.3 mM H_2_O_2_ 1 h treatment and following 24 h recovery both in the presence ad absence of 10 µM NU7026 by FACS analysis (Fig. 3D).

**Fig. 4:**
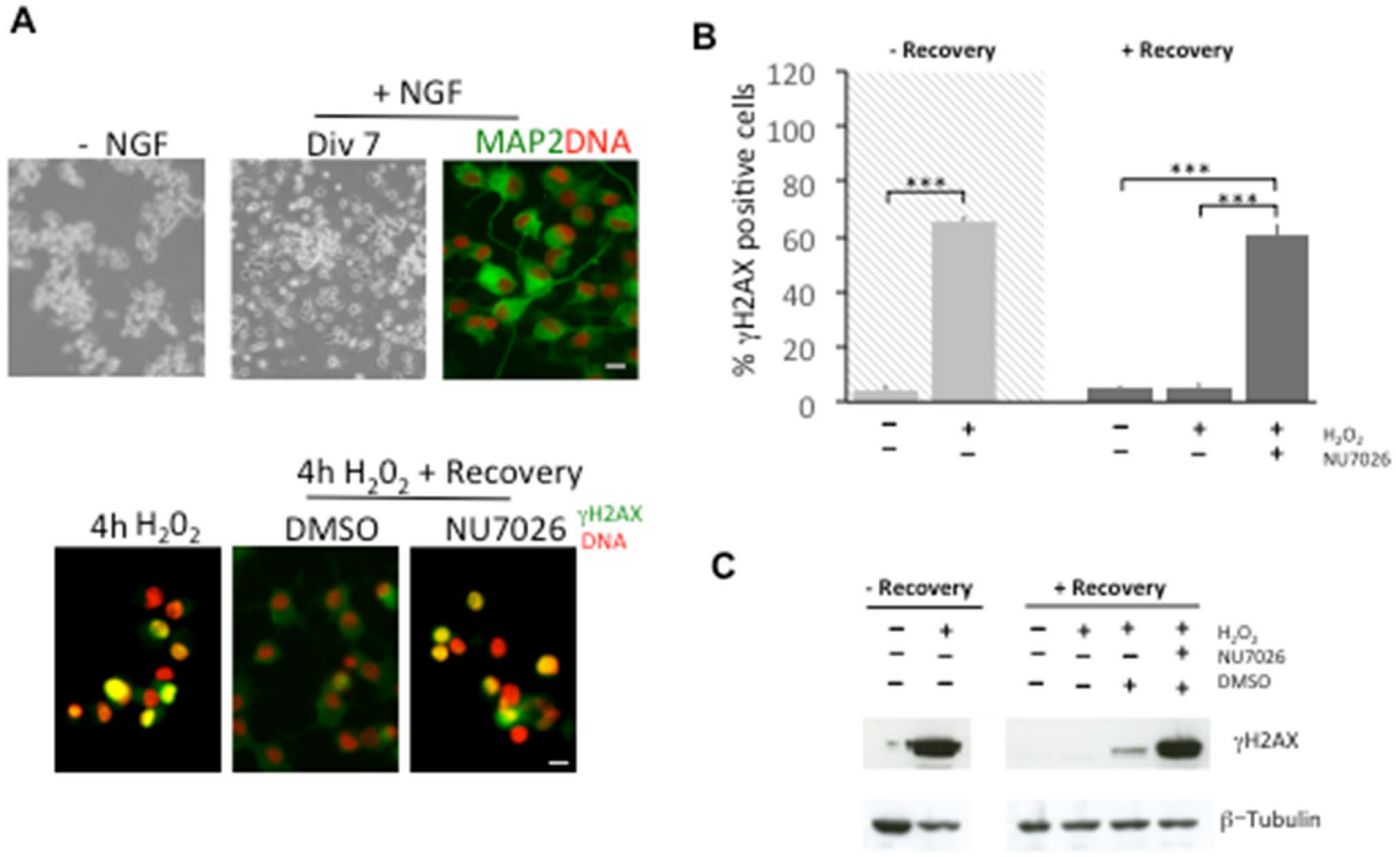
**A** Proliferating PC12 cells were differentiated with NGF for 7 days to be then exposed to H_2_O_2_ followed by a recovery in fresh medium. Cells were then fixed and immunolabelled for γH2AX foci detection. Upper panel. A microscopic field of proliferating PC12 cells in the absence of NGF showing a circular morphology and a field of PC12 cells after NGF treatment with a typical neuronal morphology, are shown in phase contrast images. After 7 days of NGF treatment, PC12 cells acquire neuronal features as indicated by the labelling with MAP2 (green) and DNA (red). Lower panel. Immunofluorescence of NGF-differentiated PC12 cells exposed to 0.3 mM H_2_O_2_ for 4 h followed by a 24 h recovery in fresh medium. Immunolabelled cells show the presence of γH2AX foci after exposure to H_2_O_2_ (green). During recovery, differentiated PC12 cells maintain γH2AX foci (green) in the presence of NU7026 as compared to control cells. Scale bar, 5 μm. **B** Bar chart showing that in NGF-differentiated PC12 cells exposed to 0.3 mM H_2_O_2_ for 4 h followed by a 24 h recovery in fresh medium, γH2AX foci are not repaired in presence of DNA-PK inhibitor, NU7026 (+55%, vs. H_2_O_2_-treated, p = 0.015). For completeness, without recovery the effect of H_2_O_2_-damage is shown (Mdiff=61%, t(6)=40.27, p < 0.001). Bars in the plots represent mean ± S.D. of cells expressed as percentage. *p < 0.05; ***p < 0.001. **C** Representative western blots of γH2AX in differentiated PC12 cells treated for 4 h with 0.3 mM of H_2_O_2_ confirming the maintenance of DNA damage in presence of DNA-PK inhibitor during recovery. After H_2_O_2_ incubation, cells were processed to obtain whole cell extracts as described in “Materials and Methods” section. β-tubulin was used as loading control. Image is representative of 3 independent experiments. (Color figure online)

Because post-mitotic cells adopt mainly NHEJ to repair damaged DNA [37, 38], we evaluated the effect of H_2_O_2_ 4 h treatment on NGF-differentiated PC12 cells (Fig. 4), a well-known neuronal model [39–42], and γH2AX foci were counted after 24 h recovery (Fig. 4B). We found that untreated control cells showed about 4% of nuclei positive to γH2AX. 4 h of exposure to H_2_O_2_ induced foci accumulation in 65% of nuclei [t(6) = 40,278, p < 0.01]. Analysis of variance showed a significant effect of 24 h recovery on the amount of γH2AX positive cells [F(3, 12)=540.2, p < 0.01]. The presence of 10 µM NU7026 DNA-PK inhibitor, significantly decreased the ability to repair DNA damage, such that 60% of cells remained positive to γH2AX (p < 0.001), and will likely undergo cell death (Fig. 4B). Western blot analysis of protein levels of γH2AX confirmed the presence of DNA damage following 4 h 0.3 mM H_2_O_2_ treatment, that decreased after recovery only in the absence of DNA-PK inhibitor (Fig. 4 C).

Overall these experiments support an anti-apoptotic role of DNA-PK independent of its DNA repair activity in proliferating cells where different DNA repair mechanisms operate. On the contrary, in neuronal cells, where DNA damage (specifically DSBs) is mostly repaired by NHEJ, the repair activity of DNA-PK complex may play a pivotal role in cell viability.

### The anti-apoptotic function of DNA-PK under oxidative stress conditions is associated with AKT phosphorylation of Serine 473 in PC12 cells

It is known that severe DNA damage induces the activation of apoptosis and signals of phosphorylation of other proteins are activated and transduced. To study the molecular mechanism underlying the anti-apoptotic function of DNA-PK complex in proliferating PC12 cells exposed to oxidative stress, we performed western blot analysis on whole cell extracts looking at the expression of several key proteins involved in the apoptotic pathway (i.e. AKT, Caspase 3 and PARP). The serine/threonine protein kinase B (PKB), also known as AKT, is a downstream effector of phosphatidylinositol 3-kinase (PI3K) and a major regulator of a variety of cellular processes, including metabolism, transcription, anti-apoptotic, proliferation, and growth [43, 44]. Activation of AKT requires phosphorylation at two key regulatory sites as follows: Thr-308 and Ser-473, the second one within a C-terminal hydrophobic motif, leads to full activation of AKT [45, 46].

**Fig. 5:**
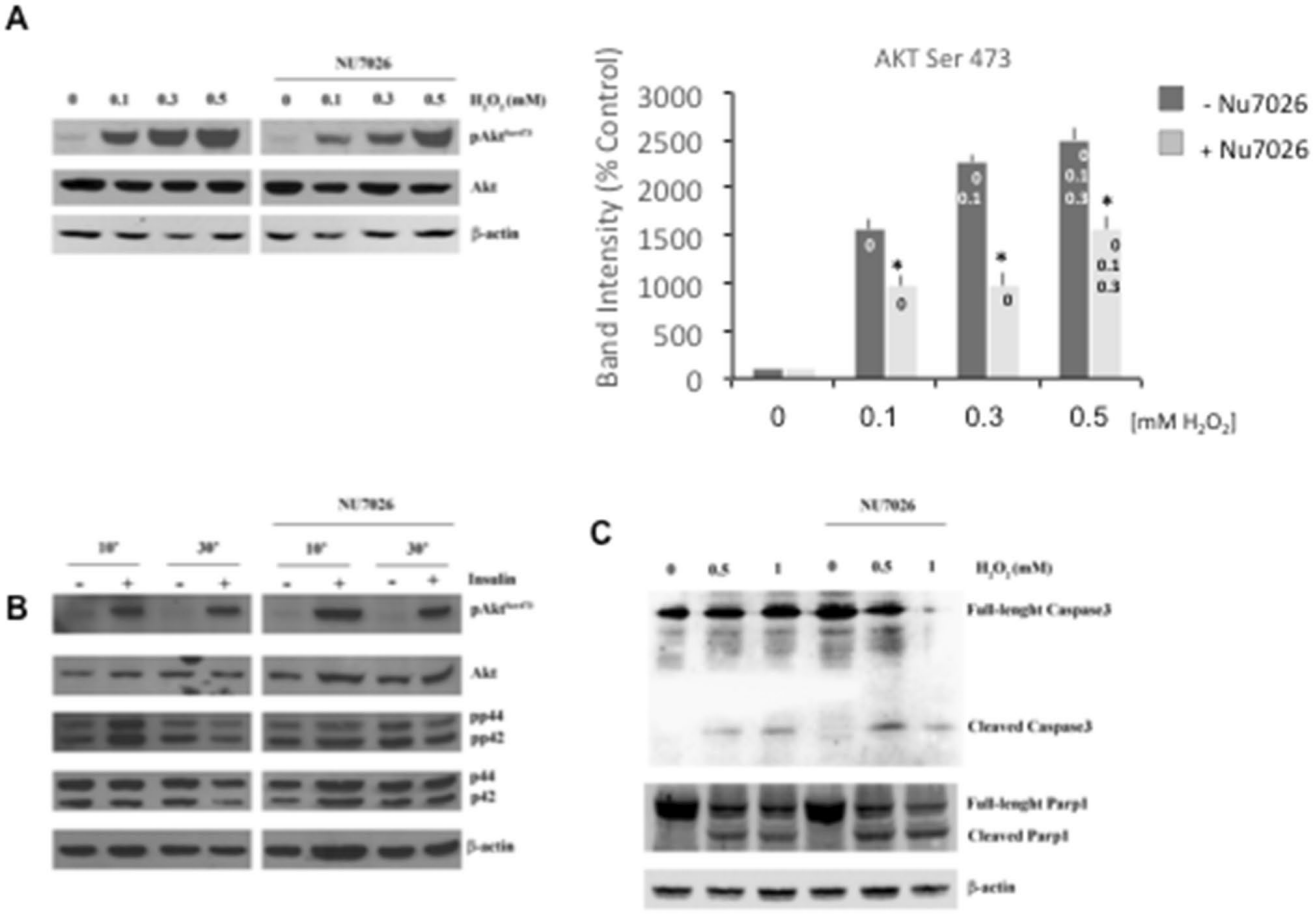
Western blot analysis of AKT phosphorylation in proliferating PC12 cells pre-exposed for 24 h with 10 µM NU7026 and then incubated for 30 min with different doses of H_2_O_2_ (**A**) or for 10 and 30 min with 100 nM insulin (**B**). **C** Analysis of full length and cleaved Caspase-3 and cleaved-PARP-1 protein levels in PC12 cells pre-treated for 24 h with NU7026 and for 30 min with 0.5 and 1 mM H_2_O_2_. Results were representative of 5 independent experiments. [*] Significant differences (p < 0.05) between NU7026 conditions within each concentration. [Concentration labels on bars] Significant differences (p < 0.05) between concentrations (0, 0.1, 0.3, 0.5) in the same NU7026 conditions, i.e. conc. labels are reported on a bar when a contrast between that group and any on its left is significant

We compared AKT phosphorylation pattern of PC12 cells treated with increasing H_2_O_2_ concentrations, ranging from 0.1 to 0.5 mM, without pre-treatment with NU7026 or with a 24 h incubation with 10 µM NU7026 (Fig. 5A). To this aim a two-way ANOVA was conducted on protein phosphorylation by NU7026-treatment and by H_2_O_2_ concentrations. Significant interactions were found for Ser-473 [F(3, 24)=3.43, p < 0.001]; successively post hoc tests were run. As shown in Fig. 5A, western blots of whole cell extracts of PC12 cells in the absence of NU7026 pre-treatment showed that all the H_2_O_2_ tested concentrations are capable to induce a strong increase of phosphorylated Ser-473AKT levels (25 fold as compared to control cells treated with 0.5 mM H_2_O_2,_ p < 0.001), while no significant alteration was observed for the phosphorylation in Thr-308 (data not shown). Differently, western blot analysis of protein extracts from NU7026 pre-treated PC12 cells 24 h before H_2_O_2_ exposure, showed a strong reduction of AKT phosphorylation of Ser-473 (38% reduction NU7026 treated cells at 0.1 mM H_2_O_2_; 57% reduction NU7026 treated cells at 0.3 mM H_2_O_2_; 39% reduction NU7026 treated cells at 0.5 mM H_2_O_2_, p < 0.01 in all cases). These results suggest a major anti-apoptotic role of DNA-PK that involves AKT phosphorylation in Ser-473. To verify the specificity of DNA-PK function in AKT regulation after oxidative stress, we tested another stimulus known to induce Ser-473 AKT phosphorylation such as insulin treatment. Cells were exposed to insulin treatment (100 mM) for 10 or 30 min and whole cell extracts analyzed for Ser-473 AKT phosphorylation in absence or presence of DNA-PK inhibitor. As shown in Fig. 5B, insulin induces Ser-473 AKT phosphorylation (both after 10 and 30 min treatment) which is not affected by DNA-PK kinase activity inhibition, indicating that DNA-PK is not involved in the activation of downstream effector AKT following insulin treatment. Similarly, DNA-PK, upon insulin treatment, is not essential for the ERK (p44 and p42) phosphorylation activation in PC12 cells (Fig. 5B).

The induction of apoptosis is associated with activation of aspartate-specific cysteine proteases (Caspases) that are present as inactive zymogens containing an N-terminal prodomain and large and small catalytic subunits. Caspases are activated either by autocatalytic processing and/or cleavage by other caspases at internal Asp residues following a variety of death stimuli, including oxidative DNA [47].

We found that following H_2_O_2_ treatment PC12 cells undergo apoptosis mediated by the activation of Caspase-3 cleavage (Fig. 5C). Interestingly, a 24 h treatment of cells with 10 µM NU7026, a concentration that blocks DNA-PK activity, increased Caspase-3 processing (Fig. 5C), supporting a protective role of DNA-PK under oxidative stress conditions. Caspase-mediated apoptotic cell death is accomplished through the cleavage of several key proteins required for cellular functioning and survival [48], including PARP-1 whose cleavage is considered to be a hallmark of apoptosis [49]. By western blot we observed, along with Caspase-3 activation, an increase in the cleaved PARP-1 (Fig. 5C).

Thus, DNA-PK exerts an anti-apoptotic function associated with Caspase-3 processing, which is further increased when DNA-PK activity is inhibited by NU7026 (Fig. 5C).

Collectively, these experiments showed that DNA-PK-mediated anti apoptotic function, upon H_2_O_2_-dependent oxidative stress, is associated with AKT phosphorylation in Ser-473 and involves the processing of Caspase-3 signalling cascade.

## Discussion

DNA repair represents a strategy to overcome the DNA damages accumulated by the cells upon exposure to different agents. Whenever DNA damage is too extensive, the DDR pathway can trigger cellular senescence and/or apoptosis [50, 51]. Among a wide range of possible DNA lesions, oxidized bases and DNA single strand breaks (SSBs) are the most common [52]. However, DNA DSBs are the most lethal form of DNA damage which, if left unrepaired, can induce a prominent loss of genetic material and ultimately cell death. Misrepaired DSBs are also deleterious because of their capability to cause genomic rearrangements, mutagenesis and more in general genomic (or mitochondrial) instability. These events are more critical in neurons, because they have a reduced DNA repair ability and a slower rate of DNA repair compared to proliferating cells, as for other differentiated cells [53, 54].

DSBs are repaired in neurons by the NHEJ pathway that relies on the DNA-dependent protein kinase complex [55].

In this study we evaluated the role of DNA-PK in oxidative stress response by using the PC12 cell model, a well-known neuronal cell line. In particular, we studied the effect of oxidative stress on DNA-PK complex expression levels and activity both in proliferating and differentiated PC12 cells. In addition, we assessed a possible dual role of DNA-PK as repair enzyme and an anti-apoptotic factor.

We found that: (i) H_2_O_2_ treatment of PC12 cells induces up-regulation of DNA-PK complex protein levels; (ii) inhibition of DNA-PK kinase activity, by using the selective DNA-PK inhibitor NU7026, increases apoptosis without affecting DNA repair in proliferating PC12 cells; (iii) the anti-apoptotic role of DNA-PK is independent of its DNA repair activity in proliferating cells, however, in neuronal cells, where DSBs are mostly repaired by NHEJ, the inhibition of DNA-PK activity causes an accumulation of DNA damage which would probably lead to cell death; (iv) DNA-PK anti apoptotic function is associated with AKT phosphorylation in Ser-473 and involves the processing of Caspase-3 signalling cascade.

This study shows that H_2_O_2_, at biologically relevant concentrations that are found in cells during acute and chronic inflammation processes, causes a marked increase in γH2AX foci, hallmark of DNA breaks, along with a significant elevation in the expression of DNA-PKcs and its regulatory subunits Ku70 and Ku86. It is likely that the observed induction of DNA-PK complex is required to counteract oxidative stress response that would otherwise lead to DNA damage and decreased in cell viability. Further studies will be required to unveil the mechanisms inducing the rapid DNA-PK expression increase after DNA damage.

Further, we showed that PC12 cells accumulate γH2AX foci that are repaired after a 24 h recovery both in the absence and presence of the DNA-PK inhibitor NU7026, indicating that other DNA-PK independent repair mechanisms may be involved. Indeed, proliferating cells possess other DNA repair systems such as the HR that can guarantee further genome stability [56]. Remarkably, while the capacity to repair γH2AX foci is maintained in proliferating PC12 cells after 24 h recovery, inhibition of DNA-PK complex activity determines an increase in the percentage of apoptotic cells, indicating an additional role of DNA-PK as an anti-apoptotic agent.

The fact that DNA-PK might possess multiple functions is in line with other studies showing that this kinase has some unusual properties. For example, its high cellular concentration [57], its presence both in the nucleus and in the cytoplasm [58], far in excess of what is probably needed for NHEJ function.

ROS generation is known to cause DNA lesions, both SSBs and DSBs and accumulation of DNA damage may represent a great concern for cells that do not replicate, such as terminally differentiated neurons. In fact, DNA damage caused by H_2_O_2_ in neurons, that have high levels of transcription and oxidative stress, may misdirect them to re-enter cell cycle albeit unsuccessfully, which in turn can lead to accumulation of excessive DNA damage causing neuronal death. Indeed, we show that differentiated PC12 cells, likewise post-mitotic neurons, upon exposure to H_2_O_2_, are not able to repair lesions in presence of DNA-PK inhibitor and may undergo cell death. This idea is also supported by the observation that in migrating cortical neurons oxidative DNA damage is normally repaired by NHEJ and failure in the repairing machinery triggers neuronal apoptosis [59]. Our data presented here may be important in understanding the roles of DNA repair enzymes and the mechanisms maintaining genomic stability in non-proliferating cells.

Moreover, we have previously demonstrated that in PC12 cells aggregated β-amyloid impairs DNA-PK activity mainly through ROS production [34]. Hence, it is possible that exposure to oxidative injuries in the presence of of amyloidogenic proteins, such as β-amyloid in Alzheimer’s Disease and huntingtin protein in Huntington Disease, elicits neuronal cell death by inhibition of DNA-PK anti-apoptotic function, leading to neurodegeneration.

It is known that DNA-PK can regulate AKT Ser-473 phosphorylation [45, 60, 61]. Moreover, Surucu et al. demonstrated that DNA-PK phosphorylates AKT Ser-473 upon induction of DNA DSBs [62]. However, the regulation of AKT by DNA-PK under oxidative conditions remained to be established. Here we show that H_2_O_2_ treated proliferating PC12 cells, following DNA-PK inhibition, undergo apoptosis, as shown by the cleavage of Caspase-3 and its target PARP-1, and this death signalling is associated with the phosphorylation of AKT in Ser-473. Differently, DNA-PK activation following H_2_O_2_ treatment does not affect AKT phosphorylation in Thr-308. This differences in residues phosphorylation is in agreement with previous observations showing that DNA-PK directly phosphorylates AKT on Ser-473 *in vitro* and its activity in cells is potently inhibited by LY-294002 and wortmannin, two PI3K-specific inhibitors [63]. In addition, AKT phosphorylation at Ser-473 was greatly diminished in DNA-PKcs short interfering (si)RNA-treated cells, and DNA-PKcs-deficient cells [64]. Furthermore, we showed that DNA-PK is unlikely to be a physiological upstream kinase mediating AKT phosphorylation upon insulin treatment, while its kinase activity has AKT as major target under oxidative stress conditions.

Other than its essential role in NHEJ, because DNA-PK is also a critical player in cell survival/death and gene transcription, it is compelling to suggest that DNA-PK can have distinct and independent functions critical for cellular homeostasis. These different roles, that deserve further analysis, may be dependent on variations in the cell cycle status, and the abundance of DNA-PKcs in different tissues.

Oxidative stress is linked to a long diverse list of human diseases including neurodegeneration and cancer. Indeed, the inhibition of DNA-PK is a very promising target in anticancer research since the efficacy of radiotherapy and some chemotherapies, working by inducing DNA DSBs in tumor cells, can be compromised by the efficient repair of DNA damage through activation DNA-PK [65].

Oxidative stress has also been suggested as the possible cause behind the inevitable process of aging, thus, identification and understanding of the key factors responsible for DNA repair and their multiple functions can unveil potential intervention points of human pathologies caused by oxidative stress.

In conclusion, our findings provide insight into the pathophysiological mechanisms underlying oxidative stress damage and suggest innovative and effective treatments for ROS-related diseases, exploiting DNA-PK-based therapeutics and/or compounds able to activate DNA-PK function.
